# (*E*)-1-(2-Hy­droxy­phen­yl)-3-(3-meth­oxy­phen­yl)prop-2-en-1-one

**DOI:** 10.1107/S2414314625001828

**Published:** 2025-03-04

**Authors:** Farid N. Naghiyev, Tuncer Hökelek, Victor N. Khrustalev, Anna Yu Zueva, Khammed A. Asadov, Alebel N. Belay, Ibrahim G. Mamedov

**Affiliations:** a Department of Chemistry, Baku State University, Z. Khalilov Str. 23, Az 1148 Baku, Azerbaijan; b Hacettepe University, Department of Physics, 06800 Beytepe-Ankara, Türkiye; c Peoples’ Friendship University of Russia (RUDN University) Miklukho-Maklay St 6 Moscow 117198 Russian Federation; d N. D. Zelinsky Institute of Organic Chemistry RAS, Leninsky Prosp. 47, Moscow 119991, Russian Federation; e Department of Chemistry, Bahir Dar University, PO Box 79, Bahir Dar, Ethiopia; University of Aberdeen, United Kingdom

**Keywords:** crystal structure, carboxyl­ate, hydrogen bond

## Abstract

In the title compound, C_16_H_14_O_3_, the phenyl rings are oriented at a dihedral angle of 3.82 (3)° and an intra­molecular O—H⋯O hydrogen bond closes an *S*(6) ring. In the crystal, weak C—H⋯O and C—H⋯π hydrogen bonds and aromatic π–π stacking occurs. A Hirshfeld surface analysis of the crystal structure indicates that the most important contributions for the crystal packing are from H⋯H (48.2%), H⋯O/O⋯H (20.0%), H⋯C/C⋯H (16.5%) and C⋯C (12.7%) inter­actions.

## Abstract 2

The title compound contains two almost coplanar phenyl rings and an intra­molecular O—H⋯O hydrogen bond.

## Graphical abstract

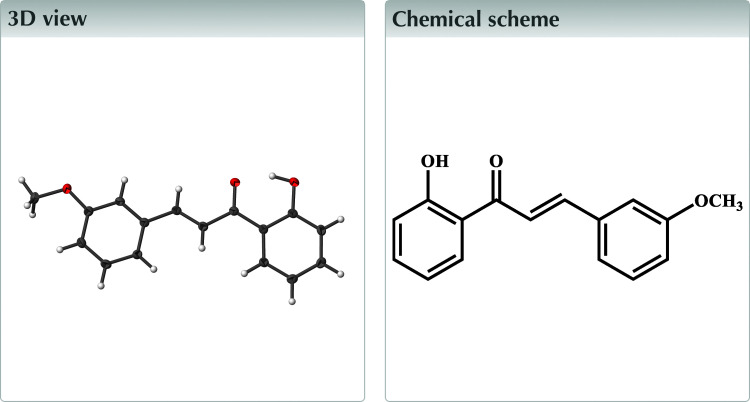

## Structure description

Chalcones are open-chain flavonoids, widely recognized as versatile building blocks in organic synthesis, owing to their α,β-unsaturated carbonyl system that facilitates the formation of diverse chemical frameworks (*e.g.*, Khalilov *et al.*, 2022 ▸). As part of our ongoing studies in this area, we now report the synthesis and structure of the title compound, C_16_H_14_O_3_, (**I**).

**Figure 1:**
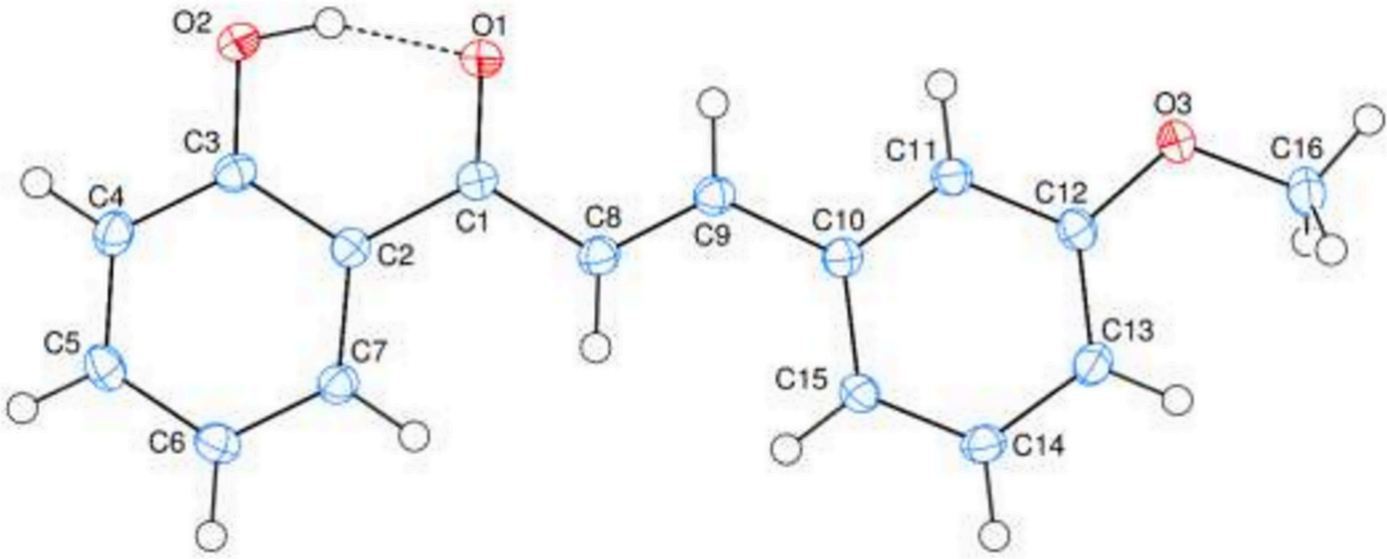
The mol­ecular structure of (**I**) showing 50% probability ellipsoids. The intra­molecular O—H⋯O hydrogen bond is shown as a dashed line.

The phenyl rings (C2–C7 and C10–C15) are oriented at a dihedral angle of 3.82 (3)° (Fig. 1 ▸) and the C2—C1—C8—C9 and C1—C8—C9—C10 torsion angles are −177.00 (9) and −178.67 (9)°, respectively. A short (and presumably strong) intra­molecular O2—H2⋯O1 hydrogen bond occurs (Table 1 ▸).

**Figure 2:**
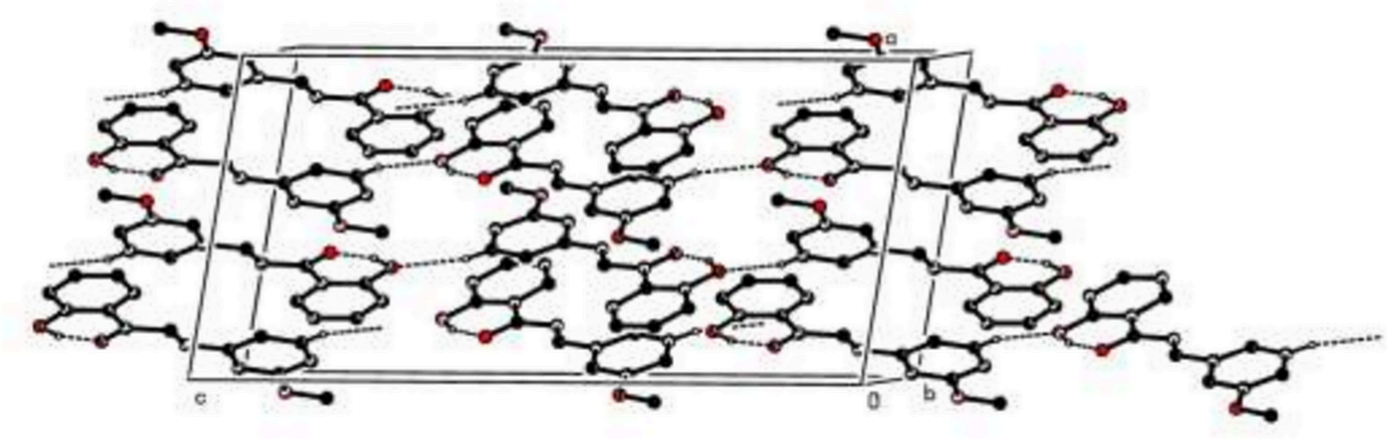
The packing diagram of (**I**) viewed down the *b*-axis direction. Intra­molecular O—H⋯O and inter­molecular C—H⋯O hydrogen bonds are shown as dashed lines. H atoms not involved in these inter­actions have been omitted for clarity.

In the crystal, weak C—H⋯O hydrogen bonds link the mol­ecules into infinite chains propagating along the *c*-axis direction (Fig. 2 ▸). Further, there are π–π inter­actions between the almost parallel phenyl rings with centroid-to-centroid distances of 3.7124 (6) Å, where the dihedral angle between the phenyl rings is 3.83 (1)° and the slippage is 1.192 Å. A weak C—H⋯π(ring) inter­action (Table 1 ▸) is also observed.

**Figure 3:**
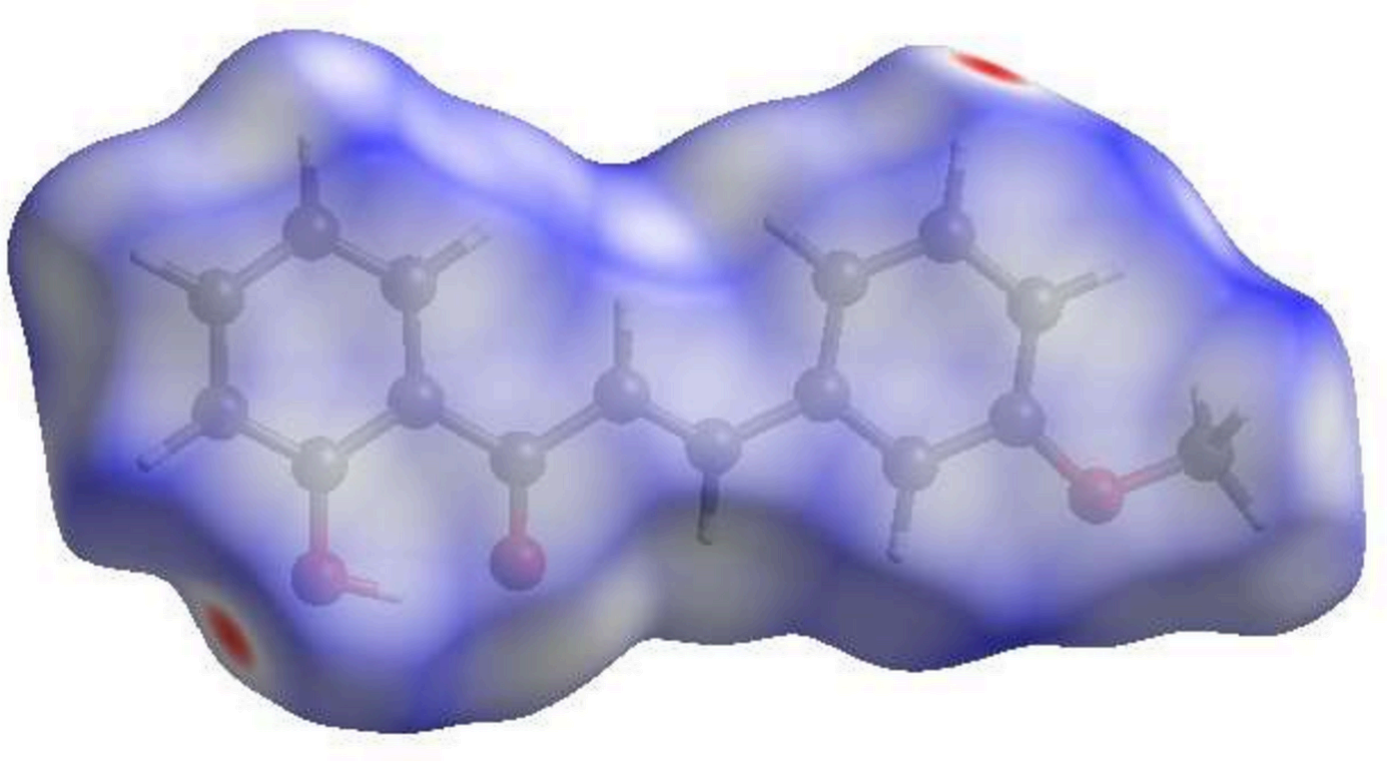
View of the three-dimensional Hirshfeld surface of (**I**) plotted over *d*_norm_.

**Figure 4:**
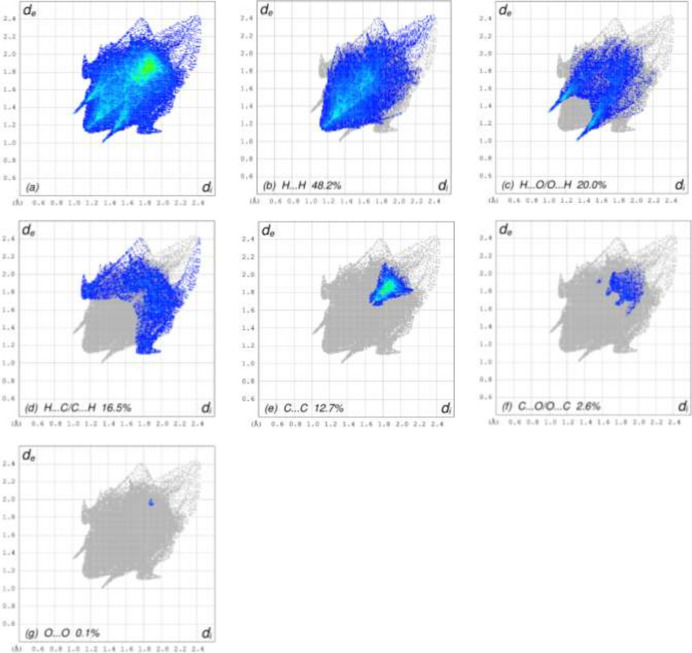
The full two-dimensional fingerprint plots for (**I**), showing (*a*) all inter­actions, and delineated into (*b*) H⋯H, (*c*) H⋯O/O⋯H, (*d*) H⋯C/C⋯H, (*e*) C⋯C, (*f*) C⋯O/O⋯C and (*g*) O⋯O inter­actions. The *d*_i_ and *d*_e_ values are the closest inter­nal and external distances (in Å) from given points on the Hirshfeld surface.

To confirm and qu­anti­fying the inter­molecular inter­actions in the crystal of (**I**), a Hirshfeld surface analysis (Fig. 3 ▸) was carried out using *Crystal Explorer 17.5* (Spackman *et al.*, 2021 ▸). The overall two-dimensional fingerprint plot, Fig. 4 ▸*a*, and those delineated into H⋯H (48.2% of the surface), H⋯O/O⋯H (20.0%), H⋯C/C⋯H (16.5%), C⋯C (12.7%), C⋯O/O⋯C (2.6%) and O⋯O(0.1%) (McKinnon *et al.*, 2007 ▸) are illustrated in Fig. 4 ▸*b*–*g*, respectively.

## Synthesis and crystallization

To a solution of 2-hy­droxy­aceto­phenone (1.36 g, 10 mmol) in ethanol (10 ml) was added 0.1 ml of piperidine as catalyst and the mixture was stirred at room temperature for 0.5 h. Then, 3-meth­oxy­benzaldehyde (1.36 g, 10 mmol) was added to the vigorously stirred reaction mixture and it was left overnight. The precipitated crystals were separated by filtration and recrystallized from an ethanol/water (1:1) solution (yield: 90%, m.p. 359 K). ^1^H NMR (300 MHz, acetone-*d*_6_, p.p.m.): 3.7 (*s*, 3H, CH_3_); 6.94 (*d*, 1H, CH, arom. ^3^*J*_H—H_ = 7.8 Hz); 7.2 (*s*, 1H, arom.), 7.3–7.4 (*m*, 5H, arom.), 7.6 (*d*, 1H, CH, ^3^*J*_H—H_ = 15.6 HZ); 7.8 (*d*, 1H, CH, ^3^*J*_H—H_ = 15.6 Hz); 7.9 (d, 1H, CH, arom. ^3^J_H—H_ = 7.7 Hz); 11.3 (s, 1H, OH). ^13^C NMR (75 MHz, acetone-*d*_6_, p.p.m.): 55.1 (CH_3_); 112.8 (CH, arom.); 115.7 (CH, arom.); 117.4 (CH, arom); 117.6 (CH, arom.); 119.3 (C_quat_, arom.); 120.1 (δbCH); 121.1 (CH, arom.); 128.8 (CH, arom.); 129.3 (CH, arom.); 135.7 (C_quat_, arom.); 136.2 (CH, arom.); 144.7 (=CH); 159.7 (C—O), 160.8 (C—O); 194.9 (CO).

## Refinement

Crystal data, data collection and structure refinement details are summarized in Table 2 ▸.

## Supplementary Material

Crystal structure: contains datablock(s) I. DOI: 10.1107/S2414314625001828/hb4506sup1.cif

Structure factors: contains datablock(s) I. DOI: 10.1107/S2414314625001828/hb4506Isup2.hkl

Supporting information file. DOI: 10.1107/S2414314625001828/hb4506Isup3.cml

CCDC reference: 2427472

Additional supporting information:  crystallographic information; 3D view; checkCIF report

## Figures and Tables

**Table 1:** Hydrogen-bond geometry (Å, °) *Cg*1 is the centroid of the C2–C7 ring.

*D*—H⋯*A*	*D*—H	H⋯*A*	*D*⋯*A*	*D*—H⋯*A*
O2—H2⋯O1	0.96 (2)	1.61 (2)	2.5156 (10)	154.9 (19)
C14—H14⋯O2^i^	0.95	2.49	3.4220 (13)	167
C16—H16*C*⋯*Cg*1^ii^	0.98	2.71	3.5097 (13)	140

Symmetry codes: (i) 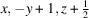; (ii) 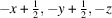.

**Table 2:** Experimental details

Crystal data	
Chemical formula	C_16_H_14_O_3_
*M*_r_	254.27
Crystal system, space group	Monoclinic, *C*2/*c*
Temperature (K)	100
*a*, *b*, *c* (Å)	10.72243 (10), 10.51268 (9), 22.22491 (18)
β (°)	99.4688 (8)
*V* (Å^3^)	2471.09 (4)
*Z*	8
Radiation type	Cu *K*α
μ (mm^−1^)	0.76
Crystal size (mm)	0.15 × 0.10 × 0.10

Data collection	
Diffractometer	Rigaku XtaLAB Synergy-S, HyPix-6000HE area-detector
Absorption correction	Multi-scan (*CrysAlis PRO*; Rigaku OD, 2021 ▸)
*T*_min_, *T*_max_	0.770, 1.000
No. of measured, independent and observed [*I* > 2σ(*I*)] reflections	14193, 2689, 2527
*R*_int_	0.031
(sin θ/λ)_max_ (Å^−1^)	0.639

Refinement	
*R*[*F*^2^ > 2σ(*F*^2^)], *wR*(*F*^2^), *S*	0.036, 0.099, 1.06
No. of reflections	2689
No. of parameters	178
H-atom treatment	H atoms treated by a mixture of independent and constrained refinement
Δρ_max_, Δρ_min_ (e Å^−3^)	0.26, −0.19

Computer programs: *CrysAlis PRO* (Rigaku OD, 2021 ▸), *SHELXT* (Sheldrick, 2015*a* ▸), *SHELXL* (Sheldrick, 2015*b* ▸) and *SHELXTL* (Sheldrick, 2015), *SHELXTL* (Sheldrick, 2008 ▸).

## full crystallographic data

### (E)-1-(2-Hydroxyphenyl)-3-(3-methoxyphenyl)prop-2-en-1-one Crystal data

**Table:**

C_16_H_14_O_3_	*F*(000) = 1072
*M**_r_* = 254.27	*D*_x_ = 1.367 Mg m^−^^3^
Monoclinic, *C*2/*c*	Cu *K*α radiation, λ = 1.54184 Å
*a* = 10.72243 (10) Å	Cell parameters from 10243 reflections
*b* = 10.51268 (9) Å	θ = 4.0–79.8°
*c* = 22.22491 (18) Å	µ = 0.76 mm^−^^1^
β = 99.4688 (8)°	*T* = 100 K
*V* = 2471.09 (4) Å^3^	Prism, colourless
*Z* = 8	0.15 × 0.10 × 0.10 mm

### (E)-1-(2-Hydroxyphenyl)-3-(3-methoxyphenyl)prop-2-en-1-one Data collection

**Table:**

Rigaku XtaLAB Synergy-S, HyPix-6000HE area-detector diffractometer	2527 reflections with *I* > 2σ(*I*)
Radiation source: micro-focus sealed X-ray tube	*R*_int_ = 0.031
φ and ω scans	θ_max_ = 80.0°, θ_min_ = 4.0°
Absorption correction: multi-scan (CrysAlisPro; Rigaku OD, 2021)	*h* = −13→13
*T*_min_ = 0.770, *T*_max_ = 1.000	*k* = −8→13
14193 measured reflections	*l* = −28→28
2689 independent reflections	

### (E)-1-(2-Hydroxyphenyl)-3-(3-methoxyphenyl)prop-2-en-1-one Refinement

**Table:**

Refinement on *F*^2^	Secondary atom site location: difference Fourier map
Least-squares matrix: full	Hydrogen site location: mixed
*R*[*F*^2^ > 2σ(*F*^2^)] = 0.036	H atoms treated by a mixture of independent and constrained refinement
*wR*(*F*^2^) = 0.099	*w* = 1/[σ^2^(*F*_o_^2^) + (0.0493*P*)^2^ + 1.7373*P*] where *P* = (*F*_o_^2^ + 2*F*_c_^2^)/3
*S* = 1.06	(Δ/σ)_max_ = 0.001
2689 reflections	Δρ_max_ = 0.26 e Å^−^^3^
178 parameters	Δρ_min_ = −0.19 e Å^−^^3^
0 restraints	Extinction correction: SHELXL, Fc^*^=kFc[1+0.001xFc^2^λ^3^/sin(2θ)]^-1/4^
Primary atom site location: dual	Extinction coefficient: 0.00040 (4)

### (E)-1-(2-Hydroxyphenyl)-3-(3-methoxyphenyl)prop-2-en-1-one Special details

**Table:**

Geometry. All esds (except the esd in the dihedral angle between two l.s. planes) are estimated using the full covariance matrix. The cell esds are taken into account individually in the estimation of esds in distances, angles and torsion angles; correlations between esds in cell parameters are only used when they are defined by crystal symmetry. An approximate (isotropic) treatment of cell esds is used for estimating esds involving l.s. planes.
Refinement. The OH hydrogen atom was located in a difference Fourier map, and refined freely. The C-bound hydrogen atom positions were geometrically placed (C—H = 0.95–0.98 Å and refined using a riding model by applying the constraint of *U*_iso_ = k *U*_eq_ (C), where k = 1.5 for methyl H atoms and k = 1.2 for the other C-bound H atoms.

### (E)-1-(2-Hydroxyphenyl)-3-(3-methoxyphenyl)prop-2-en-1-one Fractional atomic coordinates and isotropic or equivalent isotropic displacement parameters (Å^2^)

**Table:**

	*x*	*y*	*z*	*U*_iso_*/*U*_eq_	
O1	0.38451 (7)	0.58069 (7)	0.35542 (3)	0.02097 (19)	
O2	0.33559 (8)	0.41446 (8)	0.27352 (3)	0.0227 (2)	
H2	0.3655 (18)	0.489 (2)	0.2965 (9)	0.056 (5)*	
O3	0.54949 (8)	1.01783 (8)	0.60473 (3)	0.0223 (2)	
C1	0.34130 (9)	0.51250 (10)	0.39322 (5)	0.0169 (2)	
C2	0.28385 (9)	0.38836 (10)	0.37447 (5)	0.0163 (2)	
C3	0.28323 (10)	0.34535 (10)	0.31402 (5)	0.0178 (2)	
C4	0.22718 (10)	0.22920 (11)	0.29458 (5)	0.0209 (2)	
H4	0.2271	0.2010	0.2540	0.025*	
C5	0.17196 (10)	0.15544 (10)	0.33442 (5)	0.0212 (2)	
H5	0.1338	0.0767	0.3210	0.025*	
C6	0.17186 (10)	0.19577 (11)	0.39429 (5)	0.0203 (2)	
H6	0.1339	0.1445	0.4215	0.024*	
C7	0.22712 (10)	0.31026 (10)	0.41383 (5)	0.0184 (2)	
H7	0.2269	0.3370	0.4547	0.022*	
C8	0.34912 (10)	0.55674 (10)	0.45654 (5)	0.0186 (2)	
H8	0.3206	0.5031	0.4859	0.022*	
C9	0.39600 (10)	0.67174 (10)	0.47329 (5)	0.0176 (2)	
H9	0.4217	0.7228	0.4422	0.021*	
C10	0.41176 (9)	0.72683 (10)	0.53452 (5)	0.0168 (2)	
C11	0.47212 (10)	0.84450 (10)	0.54386 (5)	0.0178 (2)	
H11	0.5011	0.8858	0.5107	0.021*	
C12	0.49052 (10)	0.90231 (10)	0.60123 (5)	0.0180 (2)	
C13	0.44902 (10)	0.84144 (11)	0.64993 (5)	0.0196 (2)	
H13	0.4617	0.8797	0.6892	0.024*	
C14	0.38859 (10)	0.72374 (11)	0.64062 (5)	0.0205 (2)	
H14	0.3605	0.6822	0.6739	0.025*	
C15	0.36886 (10)	0.66657 (10)	0.58381 (5)	0.0187 (2)	
H15	0.3266	0.5870	0.5781	0.022*	
C16	0.56917 (11)	1.08037 (11)	0.66276 (5)	0.0225 (2)	
H16A	0.6094	1.1631	0.6591	0.034*	
H16B	0.6239	1.0279	0.6926	0.034*	
H16C	0.4876	1.0930	0.6764	0.034*	

### (E)-1-(2-Hydroxyphenyl)-3-(3-methoxyphenyl)prop-2-en-1-one Atomic displacement parameters (Å^2^)

**Table:**

	*U*^11^	*U*^22^	*U*^33^	*U*^12^	*U*^13^	*U*^23^
O1	0.0264 (4)	0.0192 (4)	0.0185 (4)	−0.0042 (3)	0.0074 (3)	0.0001 (3)
O2	0.0306 (4)	0.0225 (4)	0.0164 (4)	−0.0047 (3)	0.0081 (3)	−0.0007 (3)
O3	0.0296 (4)	0.0177 (4)	0.0203 (4)	−0.0056 (3)	0.0059 (3)	−0.0032 (3)
C1	0.0165 (5)	0.0172 (5)	0.0172 (5)	0.0016 (4)	0.0034 (4)	0.0010 (4)
C2	0.0163 (5)	0.0158 (5)	0.0168 (5)	0.0023 (4)	0.0029 (4)	−0.0002 (4)
C3	0.0184 (5)	0.0187 (5)	0.0171 (5)	0.0020 (4)	0.0048 (4)	0.0010 (4)
C4	0.0244 (5)	0.0205 (5)	0.0183 (5)	0.0005 (4)	0.0046 (4)	−0.0038 (4)
C5	0.0213 (5)	0.0157 (5)	0.0268 (5)	−0.0009 (4)	0.0045 (4)	−0.0032 (4)
C6	0.0210 (5)	0.0173 (5)	0.0236 (5)	0.0000 (4)	0.0066 (4)	0.0019 (4)
C7	0.0207 (5)	0.0176 (5)	0.0173 (5)	0.0012 (4)	0.0046 (4)	0.0001 (4)
C8	0.0216 (5)	0.0178 (5)	0.0168 (5)	0.0007 (4)	0.0041 (4)	0.0012 (4)
C9	0.0179 (5)	0.0179 (5)	0.0178 (5)	0.0016 (4)	0.0053 (4)	0.0006 (4)
C10	0.0169 (5)	0.0161 (5)	0.0178 (5)	0.0022 (4)	0.0040 (4)	0.0004 (4)
C11	0.0197 (5)	0.0166 (5)	0.0179 (5)	0.0005 (4)	0.0060 (4)	0.0011 (4)
C12	0.0178 (5)	0.0154 (5)	0.0208 (5)	0.0009 (4)	0.0029 (4)	−0.0005 (4)
C13	0.0227 (5)	0.0202 (5)	0.0159 (5)	0.0013 (4)	0.0033 (4)	−0.0015 (4)
C14	0.0248 (5)	0.0198 (5)	0.0179 (5)	0.0006 (4)	0.0067 (4)	0.0028 (4)
C15	0.0219 (5)	0.0148 (5)	0.0199 (5)	−0.0006 (4)	0.0052 (4)	0.0005 (4)
C16	0.0255 (5)	0.0189 (5)	0.0219 (5)	−0.0019 (4)	0.0006 (4)	−0.0048 (4)

### (E)-1-(2-Hydroxyphenyl)-3-(3-methoxyphenyl)prop-2-en-1-one Geometric parameters (Å, º)

**Table:**

O1—C1	1.2498 (13)	C8—C9	1.3384 (16)
O2—C3	1.3489 (13)	C8—H8	0.9500
O2—H2	0.96 (2)	C9—C10	1.4631 (14)
O3—C12	1.3654 (13)	C9—H9	0.9500
O3—C16	1.4321 (13)	C10—C11	1.3957 (15)
C1—C8	1.4714 (14)	C10—C15	1.4066 (14)
C1—C2	1.4737 (15)	C11—C12	1.3968 (14)
C2—C7	1.4087 (14)	C11—H11	0.9500
C2—C3	1.4165 (14)	C12—C13	1.3921 (15)
C3—C4	1.3977 (15)	C13—C14	1.3963 (16)
C4—C5	1.3815 (16)	C13—H13	0.9500
C4—H4	0.9500	C14—C15	1.3828 (15)
C5—C6	1.3967 (15)	C14—H14	0.9500
C5—H5	0.9500	C15—H15	0.9500
C6—C7	1.3797 (15)	C16—H16A	0.9800
C6—H6	0.9500	C16—H16B	0.9800
C7—H7	0.9500	C16—H16C	0.9800
			
C3—O2—H2	102.8 (12)	C8—C9—H9	116.7
C12—O3—C16	117.25 (8)	C10—C9—H9	116.7
O1—C1—C8	119.50 (10)	C11—C10—C15	119.11 (9)
O1—C1—C2	120.12 (9)	C11—C10—C9	117.96 (9)
C8—C1—C2	120.38 (9)	C15—C10—C9	122.93 (9)
C7—C2—C3	117.90 (9)	C10—C11—C12	120.91 (9)
C7—C2—C1	122.93 (9)	C10—C11—H11	119.5
C3—C2—C1	119.16 (9)	C12—C11—H11	119.5
O2—C3—C4	117.98 (9)	O3—C12—C13	124.67 (10)
O2—C3—C2	121.57 (10)	O3—C12—C11	115.69 (9)
C4—C3—C2	120.45 (10)	C13—C12—C11	119.64 (10)
C5—C4—C3	120.00 (10)	C12—C13—C14	119.47 (10)
C5—C4—H4	120.0	C12—C13—H13	120.3
C3—C4—H4	120.0	C14—C13—H13	120.3
C4—C5—C6	120.52 (10)	C15—C14—C13	121.20 (10)
C4—C5—H5	119.7	C15—C14—H14	119.4
C6—C5—H5	119.7	C13—C14—H14	119.4
C7—C6—C5	119.81 (10)	C14—C15—C10	119.66 (10)
C7—C6—H6	120.1	C14—C15—H15	120.2
C5—C6—H6	120.1	C10—C15—H15	120.2
C6—C7—C2	121.33 (10)	O3—C16—H16A	109.5
C6—C7—H7	119.3	O3—C16—H16B	109.5
C2—C7—H7	119.3	H16A—C16—H16B	109.5
C9—C8—C1	120.74 (10)	O3—C16—H16C	109.5
C9—C8—H8	119.6	H16A—C16—H16C	109.5
C1—C8—H8	119.6	H16B—C16—H16C	109.5
C8—C9—C10	126.64 (10)		
			
O1—C1—C2—C7	−176.32 (10)	C2—C1—C8—C9	−177.00 (9)
C8—C1—C2—C7	4.13 (15)	C1—C8—C9—C10	−178.67 (9)
O1—C1—C2—C3	2.66 (15)	C8—C9—C10—C11	174.88 (10)
C8—C1—C2—C3	−176.89 (9)	C8—C9—C10—C15	−5.16 (17)
C7—C2—C3—O2	−179.98 (9)	C15—C10—C11—C12	0.19 (15)
C1—C2—C3—O2	0.99 (15)	C9—C10—C11—C12	−179.85 (9)
C7—C2—C3—C4	0.32 (15)	C16—O3—C12—C13	−0.29 (15)
C1—C2—C3—C4	−178.71 (9)	C16—O3—C12—C11	179.57 (9)
O2—C3—C4—C5	−179.76 (10)	C10—C11—C12—O3	−179.36 (9)
C2—C3—C4—C5	−0.04 (16)	C10—C11—C12—C13	0.50 (16)
C3—C4—C5—C6	−0.18 (17)	O3—C12—C13—C14	179.34 (10)
C4—C5—C6—C7	0.11 (17)	C11—C12—C13—C14	−0.51 (16)
C5—C6—C7—C2	0.18 (16)	C12—C13—C14—C15	−0.16 (16)
C3—C2—C7—C6	−0.39 (15)	C13—C14—C15—C10	0.86 (16)
C1—C2—C7—C6	178.60 (9)	C11—C10—C15—C14	−0.86 (15)
O1—C1—C8—C9	3.45 (16)	C9—C10—C15—C14	179.18 (10)

### (E)-1-(2-Hydroxyphenyl)-3-(3-methoxyphenyl)prop-2-en-1-one Hydrogen-bond geometry (Å, º)

*Cg*1 is the centroid of the C2–C7 ring.

**Table:**

*D*—H···*A*	*D*—H	H···*A*	*D*···*A*	*D*—H···*A*
O2—H2···O1	0.96 (2)	1.61 (2)	2.5156 (10)	154.9 (19)
C14—H14···O2^i^	0.95	2.49	3.4220 (13)	167
C16—H16*C*···*Cg*1^ii^	0.98	2.71	3.5097 (13)	140

Symmetry codes: (i) *x*, −*y*+1, *z*+1/2; (ii) −*x*+1/2, −*y*+1/2, −*z*.
